# Pyoderma Vegetans: A Case Report in a Child Suspected to Primary Immunodeficiency and Review of the Literature

**Published:** 2015-07

**Authors:** Mahboubeh Mansouri, Azadeh Rakhshan, Mohammad Shahidi-Dadras, Abdollah Karimi, Samin Alavi

**Affiliations:** 1 Department of Immunology and Allergy, Mofid Children Hospital, Shahid Beheshti University of Medical Sciences, Tehran, Iran;; 2 Departement of Pathology, Shohada-e-Tajrish Hospital, Shahid Beheshti University of Medical Science, Tehran, Iran;; 3 Skin Research Center, Shohada-e-Tajrish Hospital, Shahid Beheshti University of Medical Science, Tehran, Iran;; 4 Department of Pediatric Infectious Diseases, Mofid Children Hospital, Pediatric Infections Research Center, Shahid Beheshti University of Medical Sciences, Tehran, Iran;; 5 Pediatric Congenital Hematologic Disorders Research Center, Mofid Children Hospital, Shahid Beheshti University of Medical Sciences, Tehran, Iran

**Keywords:** Skin disease, Pyoderma, Diagnosis, Child, Immunologic deficiency syndrome

## Abstract

Pyoderma vegetans (PV) is a rare inflammatory disorder characterized by vegetating pustules and plaques affecting the skin and mucosal membranes. It is believed that this entity is mostly associated with inflammatory bowel disease (IBD), chronic malnutrition, human immunodeficiency virus (HIV), malignancies, and other immunocompromised states. Pyoderma vegetans occurs more commonly in young and middle-aged adults. There is no sex predilection for this entity. The lesions could heal spontaneously, but usually recur and become chronic.

Our patient was an 11-year-old girl suspected to have primary combined immunodeficiency complicated by chronic recurrent vegetating pustular lesions on the face and postauricular area since one year of age. The histological features of the lesions were consistent with pyoderma vegetans. This is the first case of PV beginning from early infancy in the setting of primary immunodeficiency and in an unusual location.

## Introduction

Pyoderma vegetans (PV) is a very rare, chronic skin disease, which is identified by significant exudative, verrucous plaques with distinct and elevated margins.^1^ This entity was first reported by Hallopeau in 1898.^2^ Skin lesions generally affect the face, scalp, axilla, genitalia; and less likely the abdomen, trunk and other parts of the body.^3^

McCarthy, later in 1949, reported of some patients having PV accompanied by oral mucosal lesions. Since then, these two conditions have been considered as a single clinicopathologic entity; “pyodermatitis-pyostomatitis vegetans” (PD-PSV).^4^^,^^5^ It is difficult to treat a condition which typically affects middle-aged men and commonly is believed to be linked with ulcerative colitis. PV also has been described in different immunological abnormalities such as leukemia, lymphoma, malnutrition, HIV infection, and primary immunodeficiency disorders. There is an assumption that abnormal inflammatory reaction in immunocompromised individuals plays a causative role in this disease.^3^^,^^6^^-^^8^ Its appearance in immunosuppression states may be due to severe bacterial colonization or epithelial invasion, nonetheless, immunocompetent patients also might be infrequently affected.^6^^,^^9^^,^^10^ Herein, we report the first case of longstanding pyoderma vegetans in terms of age and location in a child with primary immunodeficiency.

## Case Presentation

**Figure 1:**
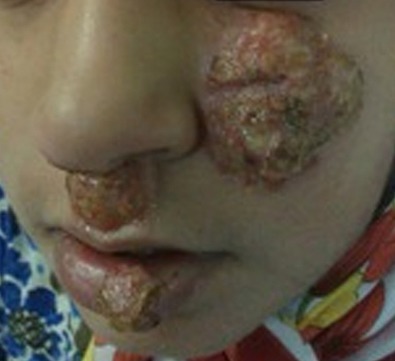
Facial lesions of PV in our patient at the age of 11 years.

An 11-year-old girl was referred to our department with a history of labial herpes infection, recurrent episodes of suppurative otitis media and suspected immunodeficiency for the past three years. Her past medical history was positive for skin eczema and recurrent chronic ulcers on the face since one year of age and repeated hospitalizations for pulmonary infections, anal abscess, and adenoidectomy since early childhood. Drug history was negative for any halogenic drug consumption. On physical examination and during the regular follow up visits, a group of well defined exudative vegetative ulcers with numerous pustules and elevated margins, exacerbating and remitting over the face, mainly around the nose, upper lip and cheek measuring from 2 to 8 cm in maximum diameter was noted (figure 1).

**Figure 2:**
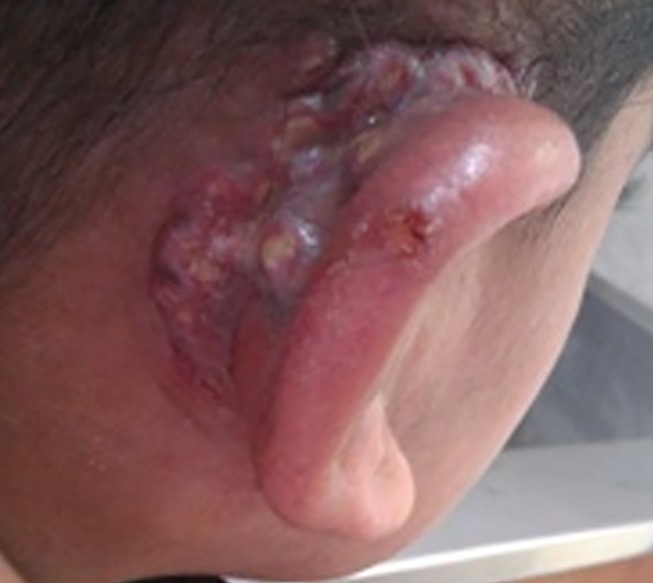
Recurrence of PV in the postauricular area in the same girl after 2 years.

**Figure 3:**
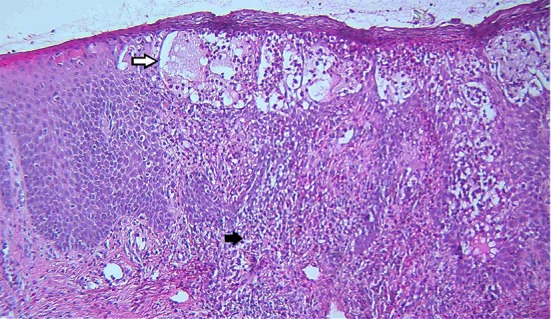
Histopathologic examination of postauricular skin biopsy: Hyperplastic epidermis with severe eosinophilic spongiosis and spongiotic vesicle formation and mixed inflammatory infiltrate with many eosinophils in the papillary dermis (Hematoxylin-eosin stain, ×100).

**Figure 4:**
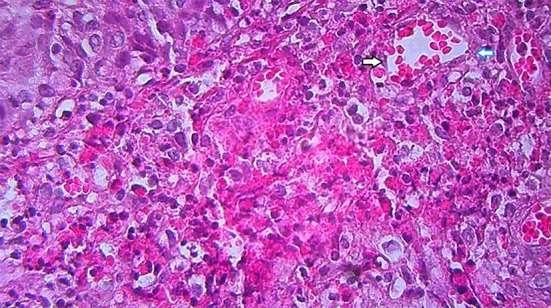
Eosinophilic dermal abscess formation (Hematoxylin-eosin stain, ×400).

At first, the lesions used to respond to a broad spectrum of antibiotics and acyclovir, however, two years later, similar vegetative ulcers appeared in the postauricular area. On examination, the ear lobe was severely inflamed and a purulent discharge from external ear canal was noticeable (figure 2). Mild coarse facies, submandibular lymphadenopathies and eczematous, dry skin specially over the elbows were also detected during the recurrence of the disease. Routine blood tests showed raised erythrocyte sedimentation rate equal to 75/1st hr, mild lymphopenia (WBC: 13.5x10³/µl with 8% lymphocytes), and marked eosinophilia (11 to 35% of white blood cells). Immunological profile was as follows: Nitroblue tetrazolium test was 99%, which was interpreted as normal. Immunoglobulin G, M, A and E (IgG, IgM, IgA and IgE) levels were 2550, 18, 29 mg/dl and 127 IU/ml, respectively. Specific antibody response to diphtheria and tetanus were 0 and 0.01, respectively. Peripheral blood cells flow cytometry showed CD3: 53%, CD4: 25%, CD8: 28%, CD19: 22%, CD16+56: 11%, and CD4/CD8: 0.89 .The results of lymphocyte transformation test (LTT) for phytohemaglutinin (PHA/mitogen) disclosed stimulation index (SI) of 10.05 for patient and 10.67 for control with normal range of 10-20. The lymphocyte SI with Bacille calmette guerin (BCG) and Candida antigens illustrated 1.14 for patient comparing to 5.37 for control and also 1.19 for patient contrasting 7.07 for control, correspondingly with the normal range of 3-10, which were severely impaired. HIV serology was negative. According to clinical features and laboratory data in favor of primary immunodeficiency, parenteral immunoglubolin (IVIG) therapy was started. Microbial culture of the skin lesions was positive for coagulase negative staphylococci. Tissue Polymerase Chain Reaction (PCR) was positive for Ctomegalovirus (CMV) and Herpes Simplex Virus Type 1 (HSV-1), but negative for Herpes Simplex Virus Type 2 (HSV-2). It was also negative for acid fast bacilli and fungi. Tzanc smear on the ulcers were positive for HSV. RIDA allergy screen food panel (R-Biopharm, Darmstadt, Germany) was negative. The hematological and gastroenterological assessments were in favor of neither malignancy nor inflammatory bowel disease. Chest X-ray disclosed bilateral basal tubular bronchiectatic changes with peribronchial thickening. Spiral Computerized Tomography (CT) scan of the thorax demonstrated multiple bilateral lymph nodes in axillary and paratracheal areas with air trapping and peribronchial thickening in both lung fields. Abdominopelvic contrast enhanced CT scan showed increased mucosal thickening of the small bowel, small mesenteric lymphadenopathies, and bilateral mild hydronephrosis. A core biopsy of the lesion of the postauricular area was performed. The characteristic histological findings consisted of pseudoepitheliomatous hyperplasia and intraepithelial or dermal microabscess composed of eosinophils, along with focal ulceration, eosinohilic spongiosis and heavy infiltration of eosinophils, lymphocytes and neutrophils in dermis (figures 3 and 4). Periodic Acid-Schiff stain (PAS) was negative for fungi organisms. A perilesional skin biopsy was negative for direct immunofluorescence. According to the above mentioned clinical features^9^ and pathological findings the definite diagnosis of pyoderma vegetans was applied. The lesions were unresponsive to a two-month period of treatment with broad spectrum antibiotics and acyclovir following which the patient was determined to receive systemic corticosteroid therapy with unfavorable response neither to one month of oral prednisone (2 mg/kg/day) nor to a three-day course of intravenous corticosteroid pulse-therapy (30 mg/kg/day). It should be noted that a written informed consent was already obtained from the parents.

## Discussion

PV which furthermore was recognized as blastomycosis-like pyoderma or pyodermatitis-pyostomatitis vegetans is an uncommon inflammatory disorder of the skin characterized by large verrucous plaques with multiple pustules and elevated margins.^1^^,^^6^ The diagnosis of PV is based on typical clinical findings and characteristic histological hallmarks including pseudoepitheliomatous hyperplasia, epidermal and dermal neutrophilia, or eosinophil carrying epidermal or dermal abscesses.^9^ Pemphigus vegetans and deep fungal infections should be kept in mind as differential diagnosis of PV. Tuberculosis cutis verrucosa, pyoderma gangrenosum, halogenoderma, squamous cell carcinoma and sweet syndrome are the other less reported clinical entities that should be considered in cases with similar lesions.^1^^,^^3^ Herein we present a 13-year-old girl suspected of having primary combined immunodeficiency who developed recurrent multiple vegetative and exudative ulcers on the face and postauricular area since one year of age, which according to clinicopathologic criteria “pyoderma vegetans” was suggested as the diagnosis.

Considering the past history of recurrent episodes of purulent otitis media and herpetic infections, observation of bilateral pulmonary bronchiectasia in radiographies, immunoglubolin profile showed elevated level of IgG but decreased level of IgM and IgA mildly increased the level of IgE along with the weak specific antibody responses to diphtheria and tetanous vaccines, decreased number of CD3+ and CD4+T cell, and reversed level of helper T cell to Cytotoxic T cell, severely impaired function of T cell against antigens, negative HIV serology, and a positive tissue PCR for CMV and HSV, all may propose a combined primary immunodeficiency state. However, none of the findings were compatible with either of the well-known immunodeficiency syndromes including Hyper IgE syndrome. Although PV has been reported to complicate immunosuppression states due to severe bacterial colonization and epithelial invasion,^1^ this is the first report of such disorder in a child with primary immunodeficiency.

To the best of our knowledge, this is the first report of pyoderma vegetans in terms of age and location. PV may affect all age groups, but mostly has been reported in young and middle-aged adults.^4^^,^^5^ The youngest patients reported were 2 siblings; 5 and 7 years old who had inflammatory bowel disease (IBD).^11^ However, our case was the earliest ever reported since her symptoms had begun from infancy. We also noted the presence of the lesions in a rather unusual site in “postauricular area” which is the first to be reported at this location. Despite the strong association between PD-PSV and IBD in the literature,^5^^,^^6^^,^^12^^,^^13^ and even regarding PV as a highly specific marker for diagnosis of IBD,^3^ there was no evidence of either intestinal disease or oral mucosal lesion in our patient. In keeping with the literature, various infectious agents such as streptococci, klebsiella, bacteroides, enterococci, pseudomonas aeruginosa and trichophyton mentagrophytes are accused to have some degrees of contribution in the development of PV.^5^^,^^10^^,^^14^^,^^15^ However, in our case cytomegalovirus, herpes simplex virus type 1 and coagulase negative staphylococci were discovered from the skin lesions, which displayed a different pattern of infection from those already have been reported.

Treatment should be specified for each patient and aimed at treating the underlying disease.^13^ Systemic corticosteroid is believed to be the first-line treatment and response to this therapy is generally favorable,^8^ while we noticed a quite poor response to the systemic corticosteroid therapy. This finding may emphasize on the necessity of having a competent immune system in order to overcome inflammation,^10^^,^^15^ whereas our patient lacked this ability due to the probable immunodeficiency and it could also be explained by a poor blood supply in a region like postauricular area. We also uncovered a marked peripheral blood and tissue eosinophilia in our case. Although eosinophilia is an important support for the diagnosis of pyoderma vegetans, but this amount of eosinophilia (up 35% of the white blood cell count) is the first time to be reported in the literature.^11^ Since eosinohils are a powerful source of proinflammatory and toxic mediators such as basic protein, lipid mediators, cytokines and a range of eosinophil proteases,^16^^,^^17^ we can also propose that the presence of a high number of these inflammatory cells might have an essential role in the initiation and perpetuation of tissue damage in our patient.

In conclusion, a 13-year-old girl highly suspicious of primary combined immunodeficiency with chronic recurrent vegetative ulcers containing numerous pustules on the face and a rather unusual location since infancy was described which based on the distinctive clinical and histological findings the diagnosis of PV was suggested. It is prudent to be familiar with this vegetative pustular skin disease, as early diagnosis together with applying appropriate treatment may prevent the perpetuation of the inflammation. 
